# The Müller-Lyer Illusion in a Computational Model of Biological Object Recognition

**DOI:** 10.1371/journal.pone.0056126

**Published:** 2013-02-15

**Authors:** Astrid Zeman, Oliver Obst, Kevin R. Brooks, Anina N. Rich

**Affiliations:** 1 Department of Cognitive Science and the ARC Centre of Excellence in Cognition and its Disorders (CCD), Macquarie University, Macquarie Park, New South Wales, Australia; 2 ICT Centre, CSIRO, Marsfield, New South Wales, Australia; 3 Department of Psychology, Macquarie University, Macquarie Park, New South Wales, Australia; University of Leicester, United Kingdom

## Abstract

Studying illusions provides insight into the way the brain processes information. The Müller-Lyer Illusion (MLI) is a classical geometrical illusion of size, in which perceived line length is decreased by arrowheads and increased by arrowtails. Many theories have been put forward to explain the MLI, such as misapplied size constancy scaling, the statistics of image-source relationships and the filtering properties of signal processing in primary visual areas. Artificial models of the ventral visual processing stream allow us to isolate factors hypothesised to cause the illusion and test how these affect classification performance. We trained a feed-forward feature hierarchical model, HMAX, to perform a dual category line length judgment task (short versus long) with over 90% accuracy. We then tested the system in its ability to judge relative line lengths for images in a control set versus images that induce the MLI in humans. Results from the computational model show an overall illusory effect similar to that experienced by human subjects. No natural images were used for training, implying that misapplied size constancy and image-source statistics are not necessary factors for generating the illusion. A post-hoc analysis of response weights within a representative trained network ruled out the possibility that the illusion is caused by a reliance on information at low spatial frequencies. Our results suggest that the MLI can be produced using only feed-forward, neurophysiological connections.

## Introduction

**Figure 1:**
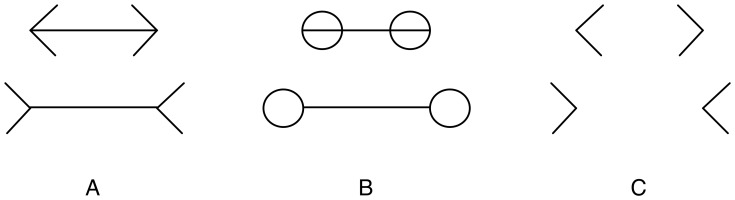
The ML illusion in various forms. A: The classical four-wing form illustrates the perceptual effect of the top line appearing shorter than the bottom line, even though the lines are of equal length. B: Terminating circles still induce a perceptual effect of line length misjudgement. C: The effect persists even when shafts are removed from the original figure.

Visual illusions have the potential to offer great insight into our visual perception. Illusions have been extensively studied by psychologists, as a method of deducing the assumptions that the brain makes and how we process visual information. One classical illusion known to induce misjudgement, is the Müller-Lyer Illusion (MLI). In the MLI, the perceived length of a line is affected by arrowheads or arrowtails placed at the ends of the line [1]. Specifically, the line appears elongated in the arrowtails and contracted with arrowheads (see Fig. 1A). Behavioural studies have shown that the strength of the illusion is correlated with factors including shaft length [2], [3], fin angle [4] and inspection time [5], [6].

Although many theories have been put forward to explain the MLI (reviewed in [7]), there is ongoing debate as to the source of the MLI. Originally, the illusion was explained as a combination of two opposing factors: ‘confluxion’ and ‘contrast’ [8], [9]. These terms were later interpreted into more modern concepts of lateral inhibition and contour repulsion [10]. Higher weighting placed on low spatial frequency information has also been investigated as a possible contributing factor towards the MLI [11], [12]. It is possible that these mechanistic explanations may not provide a full explanation of the illusion and we may need to look beyond explanations that purely involve bottom-up neural computation. Gregory was the first researcher to suggest that the images in our environment could influence our perception of the MLI and introduced another type of explanation based on misapplied size constancy scaling. Size constancy scaling refers to our visual system's ability to perceive an object as being of a constant size, even though changes in viewing distance change the size of its retinal image. To deduce the real-world size of an object, we take into account the perceived distance when scaling the retinal image size. When the depth of an image is misperceived, the scaled size judgement will also be erroneous. Gregory proposed that implicit depth cues in the arrowtails image imply that this object is more distant than the arrowheads image, such that their identical retinal sizes produce unequal perceived sizes [13].

Explaining the illusion has proven difficult because the effect persists even when the wings of the illusory figure are replaced with other terminating shapes, such as circles or squares (Fig. 1B). Even without the shaft (Fig. 1C), the perceptual effect remains. These variants demonstrate the persistence of line length misjudgment and rule out simple explanations for the cause of the illusion.

Although there is disagreement on what causes the MLI, there is some consensus on where the illusion occurs in the brain. It is commonly accepted that visual information is processed via two pathways [14]: the ventral stream or ‘what’ pathway, which extends from striate cortex to infero-temporal lobe and the dorsal stream or ‘where’ pathway, which extends from occipital to parietal cortex. A recent fMRI study shows increased blood oxygen level-dependant signal strength in the Lateral Occipital Cortex (LOC) when participants viewed the MLI versus a control image [15]. An MEG study has demonstrated results consistent with the previous fMRI data, showing strong activation along the ventral visual pathway in lateral occipital areas and the inferior temporal cortex [16]. Therefore, there is much evidence that the ventral stream plays a dominant role in processing the MLI. We hypothesised that as the MLI occurs within the ventral stream of visual processing, then a model that imitates the structure and functionality of this region should be able to demonstrate this perceptual effect.

Currently, a number of biologically plausible image recognition models exist that computationally mimic visual cortex. To date, the majority of these have been concerned with correct object identification or classification. In this paper we apply these models to a task known to produce an illusion in human observers. Here, we seek to demonstrate a similarity to human perception, not simply by reproducing a poor level of overall performance, but further by producing a specific predictable pattern of errors. We highlight several advantages for researchers from different fields who adopt this novel approach of mimicking visual ‘errors’ in computational object recognition models. For perceptual psychologists, a model that imitates illusory perception would allow for the isolation and testing of factors thought to contribute to an illusion. Errors of perception have been extremely informative in demonstrating how the human brain works. Working with a computational model opens up possibilities for conducting experiments that are difficult, if not impossible, to do in humans. These types of experiments include parameter changes (such as the level of inhibition), the modification of learning stimuli and exploration of the effect caused by artificial lesions. For the computer scientist, classification that matches human error patterns increases the biological psychological plausibility of a model. Identifying illusions can enable computers to reject interpretations of the world that yield impossible objects or paradoxes. Classification experiments may also reveal elements of neural information processing that have yet to be uncovered and lead to improved object recognition and categorisation. Thus, we can use models to test explanations of well-studied geometrical illusions from a new perspective.

This paper outlines a set of experiments conducted in HMAX, a well-established, biologically plausible model of object recognition [17]. The main goal is to analyse performance of the model in judging relative line lengths for control stimuli versus Müller-Lyer stimuli. Essentially, we want to assess whether a feed-forward object recognition model, with no exposure to natural images, can ‘perceive’ the MLI. We found a consistent pattern of errors that demonstrated a Müller-Lyer effect in HMAX after training on a non-natural set of images.

## Methods

Our experiments required a model that was *biologically plausible*, in that it could be functionally mapped to the human visual ventral stream. A number of models currently exist which have been inspired by neurophysiology, pioneered by systems such as the Neocognitron [18] and convolutional networks [19], [20]. From these biologically plausible options, we selected the model that has demonstrated much evidence consistent with neurological and psychological data. The HMAX model, with features inspired by visual cortex [17] has not only shown results congruent with psychological and neurological experiments, but it has also made correct predictions of biological phenomena [21]. We selected a version of the HMAX model that exclusively models the ventral visual stream and has successfully demonstrated mutli-class categorisation [22].

**Figure 2:**
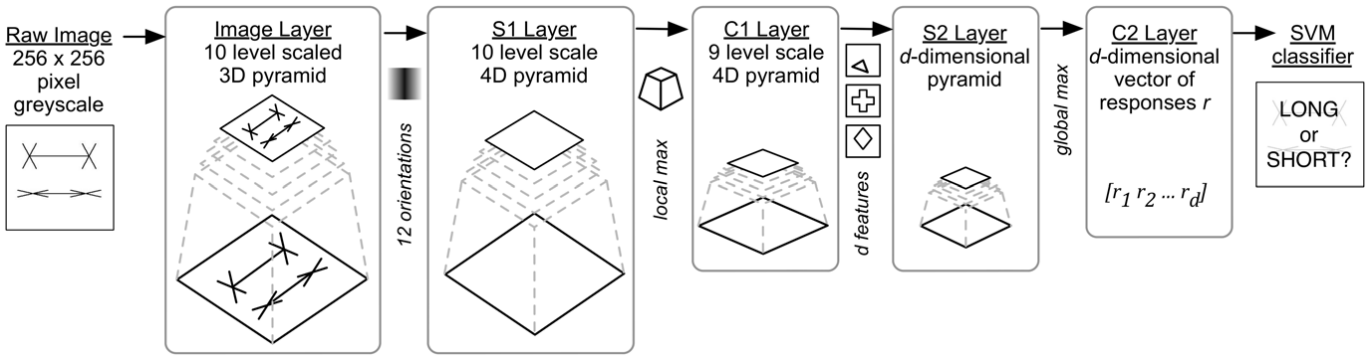
HMAX Model architecture. Information flows unidirectionally through the hierarchical layers. Input to the system is a 256×256 greyscale image and the output is a classification of the image as LONG or SHORT. The input image is first transformed onto multiple scales via the Image Layer. The following four layers alternate in their functionality, dedicated to template matching (S layers) or feature pooling (C layers). The final SVM layer performs binary classification.

The five-layer architecture setup is similar to that described in [22], where input to the network is fed through an image layer and then processing flows sequentially through the other four layers. These layers alternate in their primary functionality, dedicated to either template matching or convolution. The behaviour of these artificial cells is said to model the Simple (‘S’) and Complex (‘C’) neuronal functionality discovered by Hubel and Wiesel in cat striate cortex [23]. Simple cells demonstrate higher levels of activation in response to a specific, preferred stimulus, whereas Complex cells demonstrate invariance through high response levels across varied but related inputs. Figure 2 illustrates the set of layers within the model which are described in further detail below.

### HMAX Layer Descriptions

**Image Layer.** Input to the model is fed via the image layer, which receives a 256×256 pixel greyscale image. An image pyramid with 10 levels is constructed using bicubic interpolation, with each level 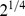 smaller than the previous. We therefore have the image duplicated at scales of 215×215, 181×181, 152×152, 128×128, 108×108, 91×91, 76×76, 64×64 and 54×54 pixels. This forms a multi-scale representation of the input image.

**S1 Layer (Gabor filter).** Output from the image layer is received by the S1 layer, which employs Gabor filters at every position and scale. Twelve orientations are used for the Gabor filters which are 11×11 in size and are applied to all levels of the 4D pyramid, before the results are normalised.

**C1 Layer (Local invariance using hard MAX).** This layer pools the response of nearby S1 units to create position and scale invariance at a local level. The range of a C1 unit forms the shape of a pyramid spanning an area 10×10 units across the base with a height of 2 levels. The response 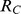 of a C unit is the maximum value of all S units 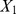 to 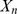 that fall within the filter range. This max filter achieves subsampling by moving around each S1 orientation pyramid in steps of five with an overlap of 2 positions and scales. The resultant C1 output is a convolved and compressed representation of S1 units. Note that the max function is not applied over different orientations, hence the C1 layer maintains a 4D pyramid structure.

**S2 Layer (Learned intermediate features).** This layer performs template matching at every position and scale in the C1 layer. A patch of C1 units centered at each position and scale is compared with a prototype patch 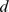. These prototypes are randomly sampled from the C1 layers of the training images in the initial feature learning stage. After feature learning is complete, each of these prototypes can now be seen as an additional convolution filter which is run over C1.

**C2 Layer (Global invariance using hard MAX).** This layer constructs a 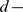
dimensional vector, where each element is the maximum response to one of the model's 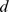 prototype patches anywhere within the image. All orientation information is collapsed into one representation. At this stage of the model, all position and scale information has been removed, so it is now a ‘bag of features’.

**SVM Layer (Decision making module).** Finally, classification of the image is performed using an all-pairs linear SVM. C2 vectors are normalised before being fed into the classifier. The majority-voting method is used to assign test images to categories.

### Task Description

**Figure 3:**
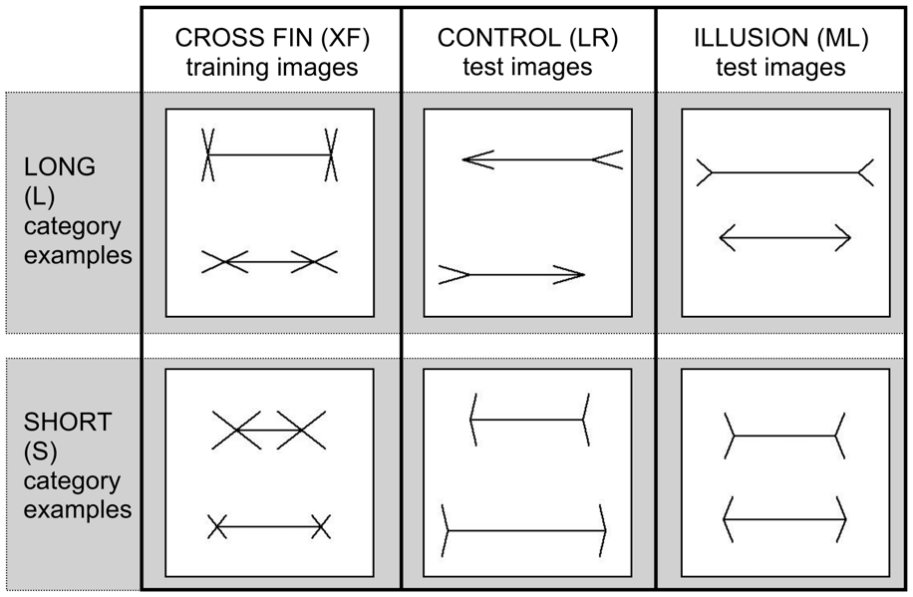
Images presented to the model, categorised as LONG (top row) or SHORT (bottom row). *Column 1*: Cross Fin (XF) images are used for training in all experiments. *Column 2*: Control (LR) images are used to test accuracy levels for a standard stimulus. *Column 3*: Illusion (ML) images are used to test performance levels for images that induce human perceptual error.

The task in these experiments was to perform a two choice category task on a set of images. This task mimics a psychophysical yes-no length discrimination procedure. The classifier had to decide whether the top line in a given image was longer (L) or shorter (S) than the bottom line. Examples of images from each category are illustrated in Fig. 3. All images fed into the model were 256×256 pixels in size, with black lines drawn using a 2×2 pixel pen on a white background. For the L condition, the top line had randomised line length between 120 and 240 pixels. For the S condition, the bottom line length was randomised also between 120 and 240 pixels. The comparator line length was randomised to be between 2 and 62 pixels shorter than the top (or bottom) line for the L (or S) condition. The vertical position of the top line was randomised between 48 and 108 pixels from the top of the image while bottom line's vertical position was randomly placed between 148 and 208 pixels. This forced the machine learner to rely on invariant properties rather than on absolute positional information for classification.

### Experimental Setup

We ran each experiment in two stages: a training stage and a test stage. The model consisted of interleaved S and C layers, with a support vector machine (SVM) on top to perform final classification (see Methods Section for details). For the training period, we exposed the network to a set of 450 images to learn features at different positions and scales. Features were only learnt in the S2 and C2 cell layers; S1 and C1 have a fixed set of features (refer to Methods Section). Once the C2 vectors were built for the training set, the SVM was trained to perform the L/S classification task. For the test phase, C2 vectors were built for the test set of images which were then classified using the SVM.

Cross Fin (XF) images (Fig. 3 Column 1) were used for training, since they contain features present in both control and test stimuli and they do not induce any illusory effects. Fin lengths were randomised between 15 and 40 pixels (measured from the end of the shaft to the tip of the fin). Fin angles were randomised between 10 and 90 degrees for both top and bottom lines. This was to prevent the classifier from relying on the end positions of fins or on bounding box information to make a length judgment. Essentially, we wanted to confirm that the machine learner was making its decision based only on the length of the inside lines (shafts) while also allowing it to be exposed to other irrelevant features.

## Results

### Experiment I: Control

The first experiment we ran was to ensure that the classifier was able to distinguish long from short images at an acceptable level of accuracy and precision for a set of control stimuli. The control stimuli we used are illustrated in Fig. 3 Column 2, where the top line has arrows pointing to the left and the bottom line has arrows pointing to the right. Fin angles were randomised between 10 and 70 degrees. We selected these control stimuli (annotated LR) because they contain the same number of features as those present in our illusion test stimuli.

**Figure 4:**
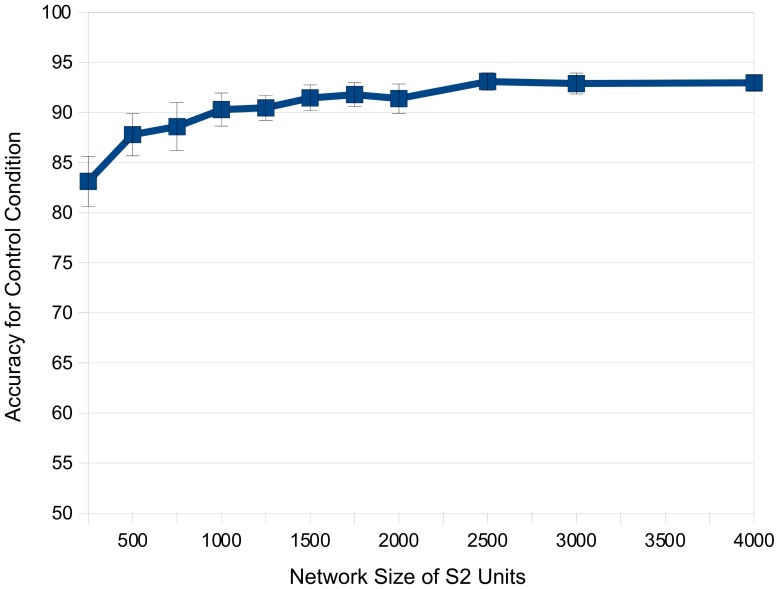
Experiment I: Control Results. Accuracy for the control condition versus the network size of S2 units. Values shown are the average of 10 runs. Error bars show standard error of the mean.

As expected, performance results for the experiment were affected by the size of the network. We varied the number of S2 units (corresponding to the number of learned features) and measured the accuracy of classification as the average of performance (% correct) in each of 10 runs with 150 test images per category. Figure 4 illustrates these results, with error bars marking standard error of the mean between runs. When the network size reached 1000 S2 cells, performance exceeded 90%. With network sizes larger than 1000, performance did not substantially improve. We therefore chose to use this network size for all subsequent experiments so as to achieve high accuracy while minimising computational expense. For our following experiments, the critical comparison was between our control and illusion conditions.

With a network size of 1000 S2 cells, we achieved an overall accuracy of 90.3% for our control. We noticed a slight bias between our LONG category (89.2%) and our SHORT category (91.47%), however this was not statistically significant (using a two-tailed paired t-test, p>0.05).

### Experiment II: Illusion Effect

The second experiment compared the results from the control experiment against those obtained using illusory Müller-Lyer (ML) images. The ML images we tested are shown in Figure 2 Column 3, where the top line always has arrowtails and the bottom line always has arrowheads. The fin length and fin angle were varied in the same way as for the control images. If the top line always has arrowtails for every single test image, the top line will appear perceptually elongated. The bottom line always having arrowheads will appear contracted. For a human observer, this means that when the two lines are objectively of equal length, the top line will appear longer. When humans are presented with any of these ML images, they will therefore classify them as ‘long’ on more occasions than when control images are used.

If the model is not susceptible to the illusion, accuracy levels should be similar to those shown in Experiment I. However, if the model is susceptible to the illusion, then we should expect to see two effects. Firstly, for the LONG category, we would expect to see the model classifying these above the accuracy level in the control condition (89.2%). Secondly, for the SHORT category, we expect to see the classifier perform worse than the control condition (91.47%). Because of the consistent configuration of the test images, the machine learner would classify images as ‘long’ more often than the control condition. This would cause it to overclassify for the LONG category and underclassify in the SHORT category.

**Figure 5:**
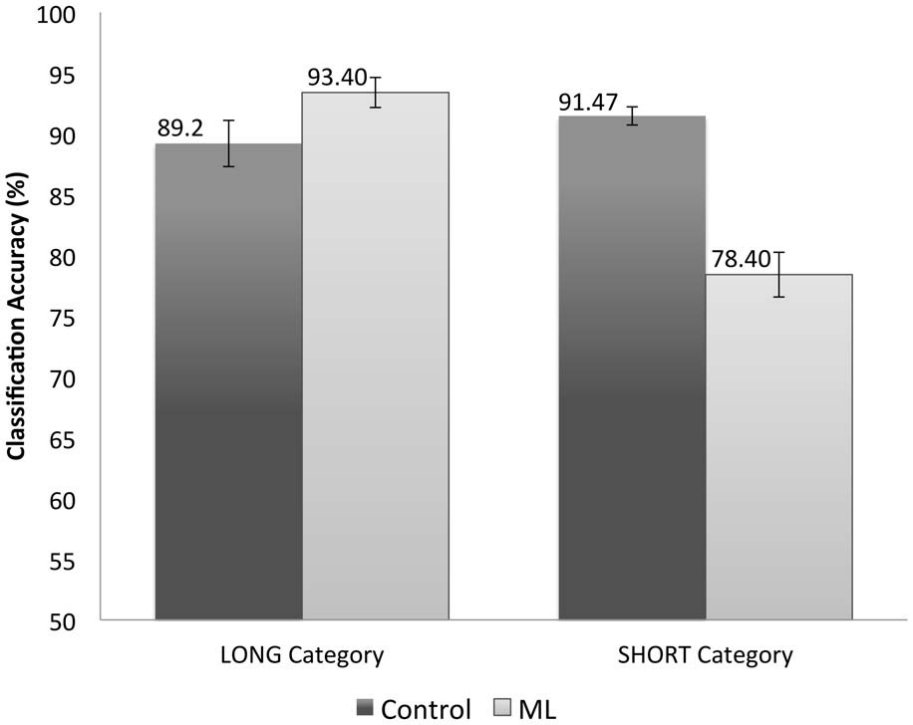
Experiment II: Control vs. Illusion Results. Accuracy (in terms of % correct) for the control versus ML images. Values shown are the average of 10 runs. Error bars indicate standard error of the mean.

Figure 5 shows the accuracy (in terms of % correct) of ML image classification plotted alongside the control condition from Experiment I. Values displayed are the average of 10 runs for 150 test images per category and error bars indicate standard error. S2 network size was set to 1000 as in the control condition. As we can see from the figure, the ML condition shows classification accuracy above the control condition for the LONG category, however this difference was only trending towards significance (using a two-sample, equal variance t-test, p = 0.0674). The inverse effect is shown in the SHORT category, where the ML condition performs under the classification accuracy of the control condition. The difference between the ML and Control conditions for the SHORT category was significant (using a two-sample, equal variance t-test, p = 0.000027). This indicates that the model is indeed susceptible to the MLI.

### Experiment III: Illusion Strength Affected by Angle

The results shown in Experiment II demonstrate errors consistent with an illusory effect; however they do not provide a detailed picture of classification performance using HMAX for control versus illusory data. We can obtain a better picture of the illusory effect within HMAX by measuring classification across incremental line length differences. By plotting classification results as a psychometric function, we are able to extract information such as the Point of Subjective Equality (PSE), for the illusory stimulus. Furthermore, we can separate out factors known to affect the strength of the illusion, such as the fin angle size or fin length, and observe consequent changes in the PSE.

**Figure 6:**
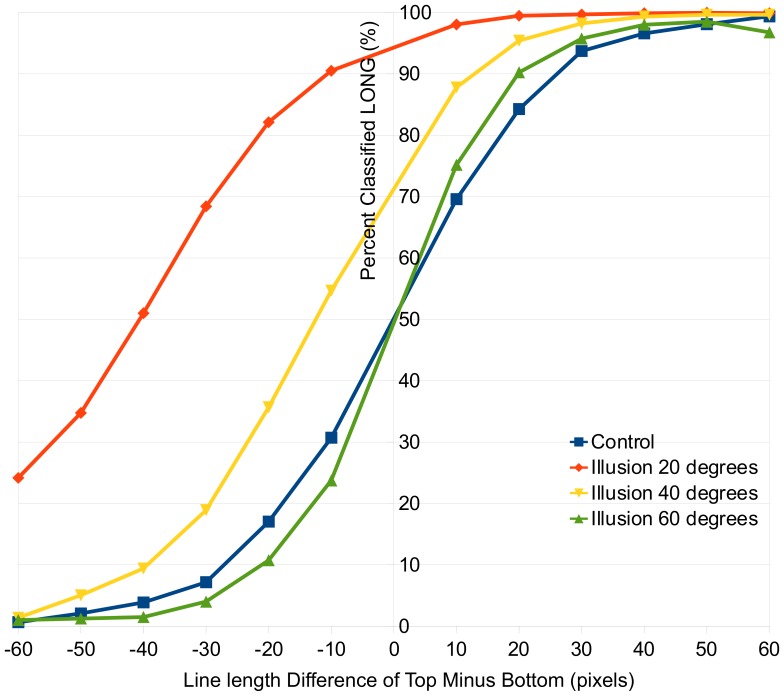
Experiment III: Illusion Strength Affected by Angle. Results here are plotted as psychometric curves with values on the left representing the SHORT condition, and values on the right representing the LONG condition. The control condition with all angles collapsed shows no bias. For illusory lines with 40 degree fins we see a PSE of approximately 12 pixels. Illusory lines with 20 degree fins show a larger PSE, congruent with human data. Illusory lines with 60 degree fins no longer demonstrate an illusory effect, indicated by intersection of the curve through 50% when the line length difference is zero.

Figure 6 shows results for the control condition versus illusion conditions with three separate fin angles, plotted as psychometric functions. Looking along the x-axis, negative values on the left indicate the SHORT category, and positive values on the right represent the LONG category. The y-axis indicates the percentage of images classified as LONG. If a classifier was always able to correctly identify the line length categories, we would see a sharp step function that takes the value of 0% on the left and 100% on the right, with a sharp transition at a line length difference of zero. Instead, what we see is a series of sigmoid functions indicating that when line length difference is large (in either negative or positive direction), it is easier for the system make a correct classification judgement. Sigmoid curves such as these are typical when mapping human psychophysical responses.

We first plotted the control condition with all angles collapsed. When there were large differences in line lengths (60 pixels), HMAX was able to categorise near ceiling for both the LONG category (far right) and the SHORT category (far left). When classification was at 50%, indicating that the top and bottom lines were judged to be the same length (i.e. the PSE), the line length difference was zero, indicating no bias. However, ML figures with 40 degree fins showed a PSE of −12.5 pixels. This indicates that with 40 degree fins, the top line must be 12.5 pixels shorter for HMAX to regard the two lines to be of equal length. Illusory lines with 20 degree angle fins demonstrated a much smaller PSE of −41 pixels. Considering human data, 20 degree angle fins would create an illusory bias of 26% [24]. For our lines of 120 to 240 pixels, this would create an average PSE of 46.8 pixels. Therefore the PSE for 20 degree angle fins in HMAX is relatively congruent with human data. Illusory lines with 60 degree angle fins no longer demonstrated an illusory effect, indicated by a PSE of zero.

Human data for the Müller-Lyer Illusion shows smaller effects as the angle becomes larger [24], which was also demonstrated by HMAX. For 20 degree data, HMAX performance matched human performance closely. However, as fin angles were increased, the illusory effect tapered off earlier in HMAX compared with human data. The 40 degree data showed a smaller effect than expected, while the 60 degree data show no effect at all, whereas humans are known to experience a Müller-Lyer effect with angles up to 80 degrees [18]. So although we observed an overall illusory effect and a degradation of illusory strength with an increase in fin angles, the illusory effect decreased faster for HMAX compared to humans.

## Discussion

In this paper, we devised a set of experiments to measure the classification performance for an ML stimulus versus a control, in a biologically plausible model of object recognition. The task was to classify images as SHORT or LONG based on the relative lengths of top and bottom lines in an image. We trained the model using a set of cross fin images that do not induce any illusion in humans and that contain all features present in test stimuli. In Experiment I, we explored different network sizes to achieve an overall classification accuracy level of 90% for our control condition. We then compared these results to an illusory stimulus in Experiment II, where we observed a respective increase and decrease in classification accuracy for the long and short conditions. This indicates that, as for human observers, this computational model of object recognition shows skewed performance levels when judging relative line lengths for Müller-Lyer stimuli. In Experiment III, we further investigated the strength of the illusion within the model by manipulating fin angle. We observed a smaller PSE for illusory stimuli with more acute fin angles, indicating a larger illusory effect. As fin angles increased, the PSE increased. This suggests that the HMAX computational model of object recognition is able to emulate the human MLI in two ways: 1) by demonstrating an overall bias in line length classification with illusory stimuli and 2) by demonstrating a larger Müller-Lyer effect with more acute fin angles.

Although HMAX is able to demonstrate an illusory effect, however, our results are not identical to patterns seen in human data. In particular, one possible reason for this is that even though HMAX is a biologically plausible model, it does omit a number of features present in the human visual system, most notably the notion of feedback or recurrent connections. Because HMAX is fundamentally a feed-forward model, to make a fair comparison between the illusion in HMAX and the illusion in humans, results from the model should be compared with human results obtained using a backward masking paradigm or repetitive transcranial magnetic stimulation (rTMS). Human psychophysical experiments performed on the MLI have, to date, not included methods that eliminate feedback processing, such as backward masking or rTMS. We plan to run further experiments using backward masking in human subjects to allow for this comparison.

Careful consideration was applied to selecting our control test stimuli. We ruled out the use of straight fin images (having wings orthogonal to the shaft) because they contain a smaller number of features compared to ML stimuli. We also ruled out the possibility of using different combinations of terminating fins because the Müller-Lyer illusion exists in many forms. We discovered that the best control stimuli were a combination of left and right arrowheads. These control images not only contain the same number of features as the ML stimuli, but also allow us to directly compare accuracy levels with varying fin angles and fin lengths.

Misclassification of the ML images, as shown in Figure 5, indicates that this computational model is susceptible to perceptual errors similar to those experienced by humans. These experimental results add to the plausibility of models that adopt a simple-complex architecture. Not only is the HMAX model able to achieve accuracy levels on par with humans in performing rapid object categorisation [25], we now show that this model can mimic aspects of human performance in misclassifying illusory stimuli.

The other significant and perhaps most surprising finding from these experiments is that the illusion was generated in a model that includes only feed-forward processing. No feedback connections are present in the HMAX model, and apart from initial feature training in the learning stage of the model, weights and connections are fixed during normal operation. Information in the system flows in one direction, from the initial image layer through simple and complex layers to the SVM. This implies that ML line length misjudgement can occur from feedforward connections alone.

However, because we do not see the MLI in its full extent under all angle conditions, this implies that, in humans, there may be other contributing factors. The gap between model and human data could be due to shortcomings within the model, such as, for example, the lack of recurrent connections. Another possibility for the mismatch between human and model data is the use of constrained training images, consisting entirely of thin black lines on a white background. Including natural scenes as part of the training set, for example, may improve the match with human data. Each of these points could be addressed separately by testing other models or by training HMAX with other image sets. Our results provide a baseline for further comparisons and the analysis of other potential explanations of the MLI.

The images used for training the model allow us to further assess proposed explanations of the MLI. The image set we used for training was inherently two dimensional in nature, consisting only of straight black lines on a white background (see XF images in Figure 3). In order to verify Gregory's misapplied size constancy scaling theory [13], we would need to train the model on images taken of 3D scenes. Gregory argues that illusory figures are ‘flat projections of typical views of objects lying in three-dimensional space’ [13]. Given that our model exhibited an illusory effect without training on any 3D images, we can be confident that misapplied size constancy scaling is not a necessary factor in causing the MLI in our model, and to the extent that this model mimics human visual processing of ML figures, it may not be necessary to explain the behaviour of human subjects. Our training image set further suggests that the ML illusion can occur in the absence of statistics of image-source relationships. Howe and Purves [26] propose that the ML illusion is caused by the “statistical relationship between retinal images and their real-world sources”. For our experiments, we did not train HMAX on any natural images and maintained a consistent number of features across our training images. Our results suggest that the Müller-Lyer illusion can be caused even without information embedded in natural images.

**Figure 7:**
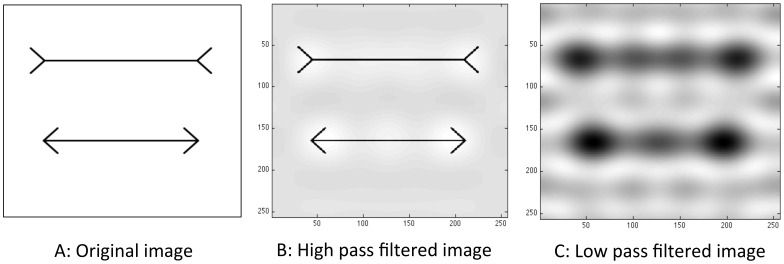
An image of the Müller-Lyer Illusion high and low pass filtered. A: the original image B: The image high pass filtered at 5 cycles per image. C: The image low pass filtered at 5 cycles per image.

**Figure 8:**
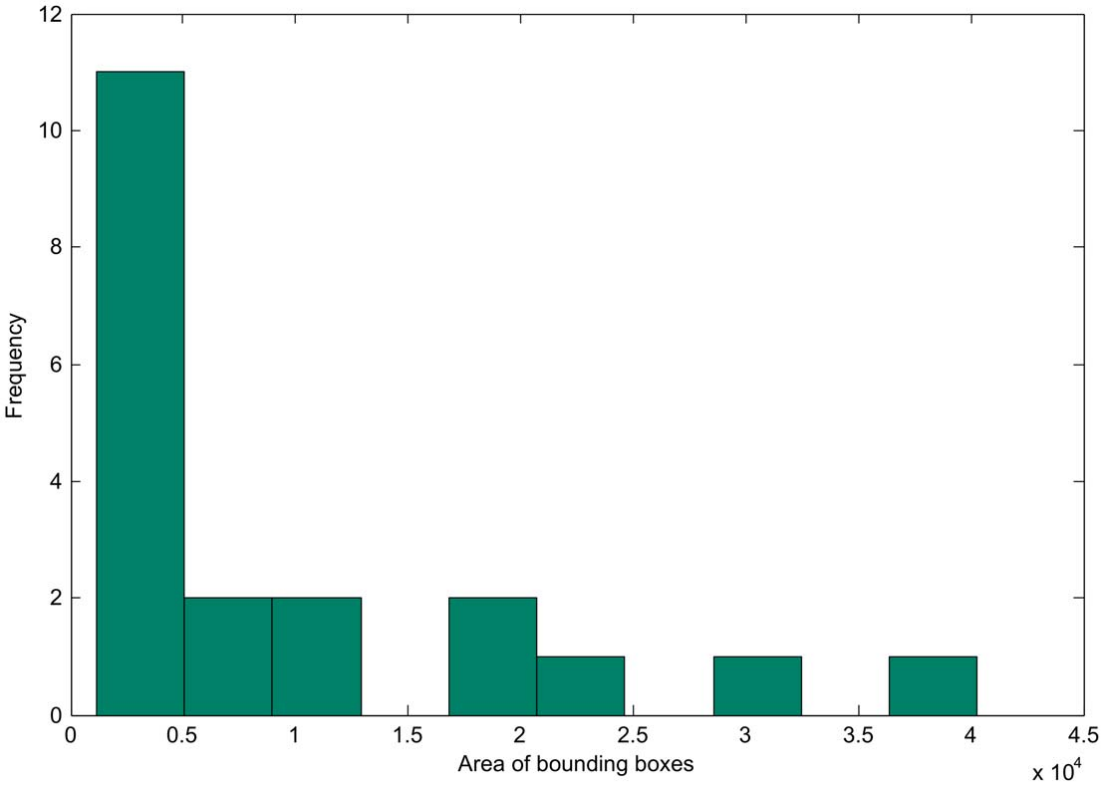
The twenty most influential features used by the SVM layer in a representative trained network, ordered by bounding box size. A post-hoc analysis of a trained network showed the 20 most influential features used to make a classification decision out of 1000 available. Stronger weighting is placed on features that have small bounding boxes and hence contain high spatial frequency information.

Ginsburg suggested that in human observers, the MLI is caused primarily by stronger weighting of low spatial frequency information [12], later supported by results from Carrasco [11]. When Müller-Lyer figures are low pass filtered, a physical difference manifests, elongating the wings-out figure (see Figure 7). If HMAX were to give stronger weighting to information flowing from units representing larger spatial scales, this would be expected to produce a similar effect. To investigate this possibility, we conducted a post-hoc analysis on one of the trained networks. We first extracted how information is weighted within the SVM layer of the model and then mapped these weights to spatial frequencies. Within the HMAX model, there is a direct relationship between spatial frequency information and receptive field size [27]. We were able to graph the bounding box sizes of the top 20 most influential features used by the SVM in order to make a classification decision (out of 1000 available). Figure 8 shows that the majority of highly weighted features fed into the SVM contain high spatial frequency information. This is inconsistent with the potential explanation that low spatial frequency information is highly influential in driving the MLI in humans. We can therefore rule out the possibility that the illusion in the network is caused by stronger weighting of low spatial frequency information.

We have demonstrated that a Müller-Lyer effect can arise in an artificial model of neural information processing. This provides an opportunity to test the extent to which hypothesised underlying neural mechanisms contribute to the illusion. For instance, lateral inhibition has been proposed as an explanation for the MLI [10]. We initially explored how changing lateral inhibition levels within the HMAX architecture affects classification performance, but altering lateral inhibition levels affected the accuracy of classification of control stimuli, which was maximal at the default parameter settings. Since we measured the Müller-Lyer effect by comparing classification performance for illusory images against control images, we therefore decided to keep the default lateral inhibition levels where the control accuracy was highest. It may be useful to further examine of the role of lateral inhibition in the future. Other possibilities include the isolation of information at different orientations to assess their relative contributions to the size of the illusion. Although beyond the scope of the current study, these have the potential to be useful tests of contributing mechanisms.

To date, there have been relatively few studies where artificial neural networks or computer models have been used to explore visual illusions [7], [28], [29]. In some cases, these artificial neural networks were not built in order to mimic neural computation, but rather to demonstrate statistical correlations in input data [29]. The model used in [29] consisted of only one hidden layer with four homogenous neurons, which few would consider to be even a crude representation of visual cortex. The work presented in [28] used a network with three hidden layers of ‘orientational neurons’, ‘rotation neurons’ and ‘line unifying neurons’. This network could roughly correspond to one layer of simple cells that provide orientation filters and one layer of complex cells that provide convolution. However, this study did not present any quantitative data and did not clearly state details of their method, such as the size or connectivity of their network. In [7], Bertulis and Bulatov created a computer model to replicate the spatial filtering properties of simple cells and convolution of complex cells in visual cortex. They compared human and model data for the Müller-Lyer Illusion, however their model centred only on filtering properties of neurons. In contrast, our study employs machine learning techniques to train the model on multiple images before running a classification task and comparing the task of interest to a control. Our study allows us to separate out the inner workings of a model from the input fed into it, in the form of training images. So although studies exist that model visual illusions within artificial neural networks, we believe that the current study represents a significant advance, being the first to model a visual illusion in a ‘biologically plausible’ artificial neural network.

That HMAX is capable of object classification, the task for which it was originally developed, may be considered impressive, given the relative simplicity of the model, which includes no feedback. However, in the current study we have presented evidence that the model is able to predict human-like performance in a completely unrelated task: that involving the discrimination of line length. Further, the correspondence of performance between man and machine represents not just degrees of classification accuracy, but also captures the pattern of errors that are made as a function of difference in line length and fin angle, and produces evidence of an illusion. These were emergent properties, rather than the model being deliberately constructed to produce these features. This might raise questions as to other visual phenomena that HMAX may be capable of accounting for, and also raises the possibility that HMAX may be capable of predicting other yet to be observed phenomena. We look forward to such research being carried out in the near future.
